# A Simple Solution for a Complex Problem: The “Sterile Cockpit” to Improve Ward Rounds

**DOI:** 10.1002/wjs.70074

**Published:** 2025-09-10

**Authors:** Ellie Treloar, Matheesha Herath, Meryl Altree, Sam Potter, Matthew Penhall, David Walsh, Lauren Kennedy, Martin Bruening, Suzanne Edwards, Jesse D. Ey, Emma L. Bradshaw, Guy J. Maddern

**Affiliations:** ^1^ Department of Surgery The University of Adelaide The Queen Elizabeth Hospital Woodville South Australia Australia; ^2^ Department of Surgery The University of Adelaide Royal Adelaide Hospital Adelaide South Australia Australia; ^3^ School of Public Health The University of Adelaide Adelaide South Australia Australia

**Keywords:** improved documentation, reduced errors, sterile cockpit, ward rounds

## Abstract

**Background:**

Ward‐round quality impacts patient outcomes, and poor conduct results in increased rates of preventable adverse events. Despite being a core component of patient outcomes, there is minimal literature informing best practice. The aviation industry has mitigated human error using a “Sterile Cockpit” to reduce interruptions and non‐essential activities. This study investigated the impact of a “Sterile Cockpit” intervention on surgical ward rounds.

**Methods:**

This prospective experimental study involved audio‐visually recording ward rounds. The intervention was a novel “Sterile Cockpit” zone involving the allocation of roles, no interruptions, one speaker at a time, invitation for nursing/allied health contribution, and “checkback” of the plan. The control group was a normal ward round. The primary outcomes were accuracy of documentation and patient satisfaction. Other outcomes included the number of parallel conversations, interruptions, and time at the bedside.

**Results:**

71 control and 70 “Sterile Cockpit” ward rounds were audio‐visually recorded. The “Sterile Cockpit” group had significantly more accurate documentation of case notes (63.6%, SD = 3.45% vs. 77.9%, SD = 3.4, mean difference 14.2, 95% CI: 4.75, 23.7, *p* = 0.003), increased nurse presence (45% vs. 68%, mean difference 0.38, 95% CI: 0.19, 0.75, *p* = 0.005), higher patient satisfaction (*p* = 0.011), reduced interruptions (mean) (0.4, SD = 0.9, vs. 0.2, SD = 0.4, IRR = 0.39, 95% CI: 0.16, 0.96 *p* = 0.039), and reduced parallel conversations (1.5 vs. 0.4, *p* < 0.001). Patient notes were completed more contemporaneously, with no additional time taken.

**Conclusions:**

The “Sterile Cockpit” is a no‐cost intervention that demonstrates improved patient and process‐based outcomes. This design is readily adaptable across specialties with the capacity to improve healthcare quality.

## Graphical abstract

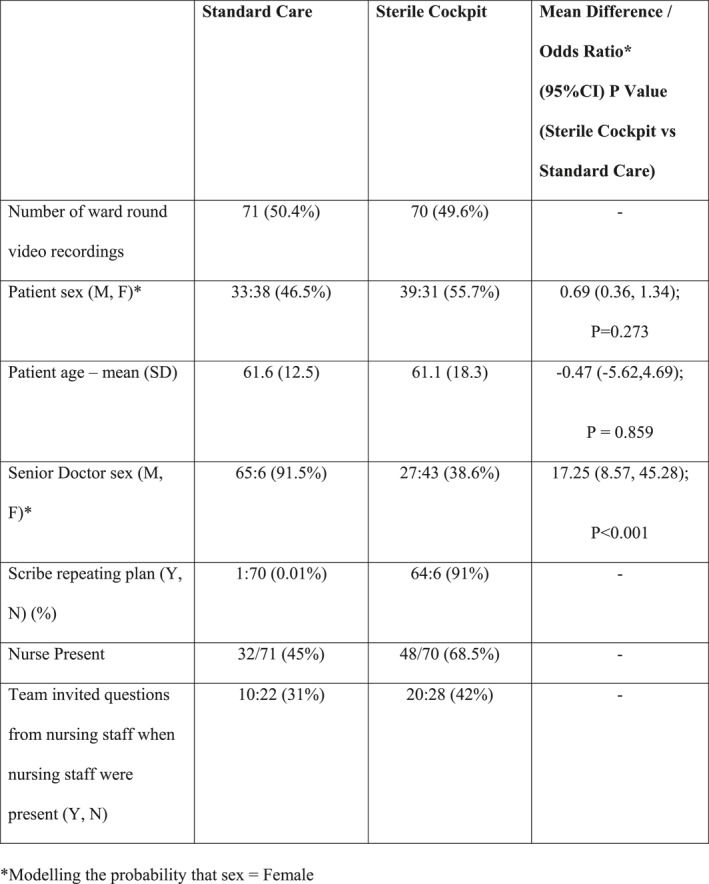

## 1 Introduction

Surgical errors represent a large proportion of adverse events in hospitals. Approximately half of these errors are deemed preventable, with many caused by breakdowns in human factors [1, 2, 3, 4, 5]. One component of surgical care known to significantly impact short‐term patient outcomes are surgical ward rounds [6, 7]. The surgical ward round is a daily routine that has existed since the advent of inpatient medicine [8, 9]. In surgical specialties, the ward round involves patient assessment, formulation of management, documentation of plans, discharge preparation, and teaching [10, 11]. Recommendations indicate that effective ward rounds should take between 8 and 15 min per patient [11, 12]. Although in practice, evidence suggests this is much lower (between 2 and 6 min) [13]. Rushed surgical rounds can result in delayed diagnosis, inappropriate management, and poor patient satisfaction [7]. This, in turn, results in preventable complications, prolonged admissions, and negative patient outcomes [1, 2, 10, 14].

In High Reliability Organizations (HROs) such as aviation, nuclear and engineering ‐ time pressure, interruptions, distractions, and hierarchical relationship issues are all human factor breakdowns that negatively impact desired outcomes [15]. However, unlike HROs—which are known to function nearly error‐free despite extremely challenging and uncertain environments, hospitals frequently experience avoidable safety incidents [16]. The World Health Organization (WHO) has adapted principles from other HROs into medicine, such as checklists and crew resource management (CRM) [15]. The use of checklists has been demonstrated to significantly improve patient outcomes in the operating room [17] and improve documentation compliance in the ward round, which has the potential to reduce adverse outcomes [18]. Despite this, significant heterogeneity exists between checklists, and their efficacy has demonstrated variable effect across studies in the ward round [18, 19, 20]. The premise of CRM is that human error is inevitable, it needs to be embraced, and it can be managed through three lines of defense: avoidance, trapping, and mitigating [21]. The “Sterile Cockpit” is a CRM concept designed to prevent crew members from performing non‐essential duties during critical moments of flight, with the purpose of focusing the attention of the crew to safe operation [22, 23, 24]. Components of the “Sterile Cockpit” such as closed loop communication and role allocation have proven efficacy in medical research, including the reduction of medication errors and interruptions [25, 26, 27]. However, the combination of all sterile cockpit concepts into a single, well defined, and reproducible intervention has not been trialled in ward rounds.

## 2 Material and Methods

This was a prospective experimental study approved by CALHN HREC (ID: 17590), registered with ANZ CTR, and conducted in the General Surgical ward at a single Australian tertiary hospital. In this study, a novel ward round intervention (Sterile Cockpit) was compared against control (Standard Care) for five outcomes: accuracy and timeliness of notes, incidence of interruptions and parallel conversations, ward round duration, presence of nursing staff, and patient engagement (measured by satisfaction and question asking).

Each ward round encounter, defined as the review of a single patient on the ward round, was audio visually recorded, and subsequently assessed for the study outcomes. Patients were eligible for inclusion and approached for consent if they were admitted to the general surgical unit, > 18 years old, and were present on the ward on the day recording was occurring. Patients were excluded if they required a translator or were held in isolation rooms requiring contact and/or respiratory precautions. All staff working in the surgical ward were invited to participate prior to the study commencement. Patients and staff were blinded to study outcomes.

Standard Care ward rounds were monitored for 4 months. Recording days were randomly selected during this time period. This was done to achieve a better representation of “Standard Care” ward rounds where the team was less expecting of every ward round encounter being recorded. Following the Standard are period, the Sterile Cockpit ward round was introduced through a staff education session.

The one‐hour in person education session consisted of a presentation on how the “Sterile Cockpit” ward round would be conducted (involving examples of the “Sterile Cockpit” in aviation), and a question/answer period. The surgical team were instructed that the “Sterile Cockpit” started and ended for each patient when declared by the team leader. The team leader's role (often the most senior member) was to enforce the “Sterile Cockpit” rules and ensure respectful, non‐judgmental, and non‐punitive conduct. The “Sterile Cockpit” was created based on aviation safety protocols and emergency resuscitation team structures. It consisted of a protected zone wherein the following rules were mandated:

1. Team roles clearly allocated prior to seeing the patient (i.e., team leader, assessor, scribe)
2. No interruptions (except for in the instance of a medical emergency)
3. Encourage nurse and allied health staff presence
4. No parallel conversations
5. At the end of the encounter, the team leader must invite:
  - team members to contribute additional information
  - nursing and allied health staff for additional information
  - the scribe to repeat the documented plan verbatim and ask for clarification if necessary.

Following the education session, staff were asked to practice the “Sterile Cockpit” for 1 week before implementation of the intervention. During this week, study investigators were present on the ward to remind the team of the “Sterile Cockpit” process and answer any questions or address concerns. In the “Sterile Cockpit” cohort, consecutive days were filmed until the desired participation number was achieved. Patients could be filmed multiple times after consenting, as the main outcomes of assessment were based on the interactions and dynamics of each specific with varying team members. This meant that if a patient was still present on the ward on the day of filming, they would be recorded again if amenable.

At the conclusion of each ward round encounter, patients completed a short survey to assess their understanding of the discussion and their satisfaction of that specific ward round encounter. This occurred in both cohorts. The survey consisted of 7 questions on a 5‐point Likert scale, 1 question on a 3‐point Likert scale, and 1 free text box to make comments. Results were tallied with a maximum possible survey score of 38. This overall score was used as a measure of patient satisfaction. Survey questions can be found in Supporting Information S1.

In order to capture audio‐visual recordings of ward round encounters between patients and their team for assessment, written informed consent was obtained from both patients and staff. Risk mitigation strategies regarding privacy concerns with recording were explained. To adhere to ethical standards, visual recordings were stored in an encrypted hard drive and de‐identified where possible. The audio‐visual recordings enabled the investigators to assess the accuracy of documentation (by comparing speech to case notes), as well as compliance with the intervention. All patients involved were formally consented one day prior to the ward round. A small video camera (GoPro) was positioned in their room prior to the ward round. Outcomes such as length of stay were assessed on a per patient basis. Patient case notes were extracted—immediately following the ward round, throughout the day, and at the cessation of the study—to determine if and when the notes were updated.

Audio‐visual recordings were independently reviewed by two research team members and transcribed verbatim (MP, SP). Transcriptions were independently assessed for accuracy by a third senior researcher (ET). Transcription enabled all items of communication to be grouped into questions or answers, and who participated in the communication, that is, patient to doctor, or doctor to doctor. The grouped transcriptions were then distributed to two senior surgeons with a cumulative experience as consultants of 32 years (LK, DW), who were blinded to the intervention and study end points, to allocate a level of importance for each communication point. These consultants were external to the team being studied. If there was discrepancy between these two reviewers, the grouped transcriptions were sent to another blinded senior surgeon consultant with 25 years of experience for resolution (MB). Definitions for level of importance were developed through a Modified Delphi Process, involving clinical and researcher staff with extensive experience in the conduct of, or research regarding, the surgical ward round. Communication points were rated either “Major”, “Minor”, or “Not Significant”, based on their importance to patient care. A “Major” point was defined as a point of discussion that would likely have a direct or serious impact on the patient's progress or delay their discharge if not documented (e.g., drain output, diet change, booking for theater). A “Minor” point was defined as a point which would cause minimal or no harm if not documented, but was still relevant to care (e.g., verbalization of decision‐making processes rather than the final decision made). Any discussion that was not relevant to the patient's care was classified as “Not significant”. Any differences of “Major”, “Minor”, and “Not significant” ratings between the two reviewers were resolved by a third reviewer.

Transcriptions were then compared to patient case notes by two independent blinded study investigators (MA, ET). If discussion points were documented in the case notes it would be classified as “Documented correctly”, and if incorrect, as “Documented incorrectly”. Omissions were classified as “Not Documented”. This allowed investigators to determine the distribution of “Major”, “Minor” and “Not significant” points discussed within each ward round encounter, and the accuracy of documentation. Additional data collection involved two blinded reviewers (SP, MP), reviewing each video for the presence of: parallel conversation, interruptions, questions asked by the patient and/or clinical staff, and the number of staff present. Results were included if the two reviewers had scores within 20% and were assessed by a third investigator (ET) if discrepancy was over 20%.

### 2.1 Statistical Analysis

Statistical analysis was performed by an experienced biostatistician (SE). Fisher's Exact Conditional Test for two Proportions with alpha of 0.05 and a 2‐sided test, determined that a sample size of *N* = 70 per group would provide 80% power. Descriptive statistics were performed for all outcomes and covariates: mean and standard deviation (SD) for normally distributed continuous or interval variables, median and interquartile range (IQR) for skewed continuous or interval variables, and frequency and percentage for categorical variables. Univariate linear regressions, as well as negative binomial regressions were performed for the predictors and outcomes: Accuracy “Major” Percent, Accuracy “Minor” Percent, Total Time Parallel Conversations, Duration in Seconds, Patient Age, Patient Sex, Nurse presence, Interruptions, Parallel Conversations, Number of People in Room, Questions Patient to Doctor, Questions Doctor to Doctor, and Questions Doctor to Patient. Additionally, bivariate linear regressions were performed to assess the relationship between the outcomes Accuracy “Minor” Percent and Accuracy “Major” Percent versus the predictor intervention and various covariates (one model at a time). The statistical software used was SAS On Demand for Academics (SAS Institute Inc. 2024 Version 9.4: Cary, NC, USA). A *p* value ≤ 0.05 was considered statistically significant.

## 3 Results and Discussion

There were 141 audio‐visual captures of ward round encounters between a patient and their care team (71 “Standard Care”, 70 “Sterile Cockpit”). These 141 encounters involved 67 different patients, who had their ward round encounter with the surgical team recorded between one to five times. There were 16 patients who declined to participate. Their reasons included: not wanting to be filmed (*n* = 6), feeling too unwell (*n* = 4), “didn't feel like it” (*n* = 3), a perception that their care was “too good and did not want to jeopardize it” (*n* = 2) and Other (*n* = 1). No staff declined participation. There were 16 senior clinicians (i.e., Consultants, Fellows, and Registrars) and 16 junior clinicians (i.e., interns) who participated. The senior clinicians led a median of 9 (IQR = 3, 14.5) ward rounds, and each intern documented a median of 4.5 (IQR = 2.3, 15.3) ward rounds. In the control group, the ward rounds were led 16 times by a consultant, 15 times by a fellow, and 35 times by registrars. In the sterile cockpit group, they were led 17 by a consultant, 17 times by a fellow and 36 times by registrars. Table 1 displays the demographics of both groups.

**TABLE 1:** Demographics.

	Standard care	Sterile Cockpit	Mean difference/odds ratio^a^ (95% CI) *p* value (Sterile Cockpit vs. standard care)
Number of ward round video recordings	71 (50.4%)	70 (49.6%)	—
Patient sex (M, F)^a^	33:38 (46.5%)	39:31 (55.7%)	0.69 (0.36, 1.34); *p* = 0.273
Patient age—mean (SD)	61.6 (12.5)	61.1 (18.3)	−0.47 (−5.62,4.69); *p* = 0.859
Senior doctor sex (M, F)^a^	65:6 (91.5%)	27:43 (38.6%)	17.25 (8.57, 45.28); *p* < 0.001
Scribe repeating plan (Y, N) (%)	1:70 (0.01%)	64:6 (91%)	—
Nurse present	32/71 (45%)	48/70 (68.5%)	—
Team invited questions from nursing staff when nursing staff were present (Y, N)	10:22 (31%)	20:28 (42%)	—

^a^ Modeling the probability that sex = Female.

The “Sterile Cockpit” significantly improved the documentation accuracy of ‘Major’ points during the Surgical ward round, increasing the mean from 63.6% (SD = 3.45) to 77.9% (SD = 3.4) (*p* = 0.003). It resulted in 61% less interruptions (mean number of interruptions per ward round control group: 0.4 (SD = 0.9) versus “Sterile Cockpit” 0.2 (SD = 0.4) (IRR = 0.39, 95% CI [0.16, 0.96]), (*p* = 0.0398)). Additionally, the number of parallel conversations reduced by 74% (average number of parallel conversations per ward round control group: 1.5 (SD = 1.6) versus ‘Sterile Cockpit’ 0.4 (SD = 0.8), (IRR = 0.26, 95% CI [0.16, 0.43]) (*p* < 0.0001). The ‘Sterile Cockpit’ intervention did not impact the documentation accuracy of “Minor” points (*p* = 0.15), the number of documentation errors “Major” (*p* = 0.90), or “Minor” (*p* = 0.71), or the time taken per ward round encounter (*p* = 0.06). Table 2 displays the impact of the “Sterile Cockpit” on each outcome. Regarding adherence to the “Sterile Cockpit” rules, there was a high uptake of the interns repeating the plan back to the team (Control: 1%, vs. Sterile Cockpit: 91%), but a smaller uptake of the team leader inviting the nursing staff for their input when they were present (Control: 31%, vs. Sterile Cockpit: 42%).

**TABLE 2:** The impact of the “Sterile Cockpit”.

Outcomes	Standard care (mean, SD, *N* (%))	Sterile Cockpit (mean, SD, *N* (%))	Mean difference/odds ratio/IRR (95% CI) (Sterile Cockpit vs. standard care)	*p* value
Group	*N* = 71	*N* = 70	—	—
“Major” points documented accurately	63.6 (22.0)	77.9 (16.6)	14.2 (4.75, 23.7)	**0.003** ^a^
“Minor” points documented accurately	51.8 (34.6)	61.6 (40.2)	9.83 (−3.70, 23.4)	0.15
Number of documentation errors (“major” points)^d^	*N*: 15	*N*: 15	1.05 (0.47, 2.35)	0.90
Number of documentation errors (“minor” points)^c^	*N*: 4	*N*: 3	0.75 (0.16, 3.48)	0.71
Prevalence of case notes updated (*N*, %)^c^	20/71 (28%)	1/70 (0.01%)	0.38 (0.19, 0.77)	**0.007**
Number of interruptions (mean) (including 0)^b^	0.4 (0.9)	0.2 (0.4)	0.39 (0.16, 0.96)	**0.039** ^a^
Number of parallel conversations (including 0)^b^	1.5 (1.6)	0.4 (0.8)	0.26 (0.16, 0.43)	**< 0.001** ^a^
Total time of parallel conversations (seconds)	33.6 (38.3)	18.5 (7.3)	−15.10 (−32.18, 1.97)	0.08
Number of people in the room^b^	5.94 (1.66)	5.21 (1.51)	0.88 (0.76, 1.01)	0.07
Nurse presence^c^	32/71 (45%)	48/70 (69%)	0.38 (0.19, 0.75)	**0.005** ^a^
Questions: from doctor to doctor^b^	5.93 (2.98)	6.86 (3.69)	1.16 (0.98, 1.37)	0.09
Questions: from doctor to patient^b^	2.93 (2.56)	4.34 (3.09)	1.48 (1.15, 1.92)	**0.003** ^a^
Questions: from patient to doctor^b^	3.03 (2.79)	3.14 (2.96)	1.04 (0.77, 1.40)	0.81
Duration (seconds)	232.7 (117.4)	271.5 (128.4)	38.8 (−1.53, 79.1)	0.06
Survey total score	28.6 (5.84)	30.9 (4.36)	2.29 (0.52, 4.04)	**0.011** ^a^

*Note:* The use of bold was used to indicate a statistically significant *p* value.

^a^ Statistically significant.

^b^ Negative binomial models with Incidence Rate Ratio and 95% CI.

^c^ Binary logistic models: Modeling the probability that Nurse presence = “No”, Number of errors minor is 1 and Updated Notes is Yes|.

^d^ Ordinal logistic models: Modeling the probability that number of errors major is high (1 or 2).

Additionally, in the “Sterile Cockpit” ward rounds, staff retrospectively updated 62% fewer case notes compared to the “Standard Care” ward round (Odds Ratio = 0.38, 95% CI [0.19, 0.77]) (*p* = 0.007). There were 20 (out of 71) case notes that were retrospectively updated, this improved the case note accuracy by an average of 43% (ranging from 14.3% to 100%). The average time taken before case notes were updated was 3 h 45 min (ranging from 55 min to 5 h 44 min). 12 of the retrospectively updated case notes occurred on a Wednesday, the day of consultant rounding. Additionally, two of the 71 patient notes were not created until after the patient was discharged, or until the following morning ward round (up to 23 h later). In the “Sterile Cockpit” cohort, only one case note was retrospectively updated (occurring 3 h and 49 min later), improving the accuracy by 14.8%. Having accurate and timely notes accessible for nurses and the allied health teams, results in care plans, orders, concerns and patient transfers to be actioned earlier. This ultimately reduces delays and improves patient flow.

Accurate and contemporaneous documentation is mandated by the Australian Health Practitioner Regulation Agency (AHPRA) and is crucial for the continuity of care between medical teams, facilitation of timely completion of tasks, and minimizing preventable errors [7, 19]. Additionally, patients report that varying and inconsistent plans are frustrating aspects of their care [28]. The “check back” component of the “Sterile Cockpit” (where the intern was asked to repeat their documentation verbatim) played a crucial role in improving the timeliness and accuracy of case note documentation. It created a safe space to identify errors or misinterpretations at the bedside, a plan agreed and understood by all and provided a teaching opportunity [29]. The clinical implications of improved documentation are reduced adverse events, shortened hospital stay, and less medicolegal risk [30, 31].

An interesting result of this study was that despite the increase in accuracy of documentation in the “Sterile Cockpit” group, the number of documentation errors was almost identical between the two groups. The hypothesis for this is that human error is inevitable, can be complex, and potentially not completely omitted.

Another fundamental component in ensuring effective information sharing, documentation and discharge planning, was increasing nurse presence [11, 28]. In this study, nurse presence significantly increased, from 45% to 68% (*p* = 0.0053). Modern medicine is multidisciplinary, and the absence of any member can delay interventions, delay treatment, worsen patient outcomes, and increase costs [11]. Nursing staff can feel invisible in the process of surgical ward rounds, and their absence (physically and/or verbally) has clear consequences on the patient's journey [28]. The “Sterile Cockpit” cohort demonstrated an increase in nurse presence. This may be due to the perception that nursing input was welcomed, or simply that the surgical team sought input from the nursing staff, thereby ensuring their presence.

Finally, patients in the “Sterile Cockpit” group demonstrated increased satisfaction with the ward round (*p* = 0.011) and were asked more questions by their doctors (*p* = 0.003) than patients in the Standard Care group. Patient satisfaction and engagement are commonly used indicators for measuring the quality of healthcare in hospitals [32, 33] and are known to reduce serious safety incidents [34, 35].

The surgical ward round is a time pressured part of the doctor's daily routine, often conducted quickly [36, 37] In other HRO's, almost 80% of incidents are cited to be due to “time compression, high workload, preoccupation” or a situation where human performance is degraded by a perceived or actual need to hurry [38]. In environments of perceived time compression, it is more likely that team members will not “speak up” regarding small errors or concerns. However, this is usually when the errors are made. Although the “Sterile Cockpit” design made no attempt to reduce the time spent on the ward round, it created an environment where focus from every member of the team was commanded, and team members felt comfortable to “speak up”. This ultimately improved several patient and process‐based outcomes, whilst adding no additional time per patient.

It is important for leaders of ward rounds to create environments of psychological safety [39, 40]. Psychological safety is the perception that the environment is safe for contributing or exposing vulnerability without fear of negative consequences [41, 42]. These environments improve information sharing, error detection, facilitation of education, and reduce the chance of human error; all of which lead to better patient outcomes [4, 41, 43]. Additionally, this promotes patient engagement and communication with healthcare providers [42]. By giving junior doctors and nursing staff a prompt to contribute, an environment of psychological safety was created, and positive results were demonstrated.

Methodological limitations that may have impacted the results of this study involve the potential “Hawthorne Effect”, the single‐site observational cohort, and sex differences of staff leading the ward round. It is possible that the “Hawthorne Effect” (modifying behavior in response to being observed) was present, as all participants were made aware of the audio‐visual recording for ethical purposes. However, as the audio‐video recording occurred in both cohorts, it is likely that this was nullified for comparison purposes. Additionally, although patients and staff were aware of the audio‐visual recordings, they were blinded to the five study outcomes. Due to the nature of the methodology, researchers were not blinded to the cohort that patients were allocated into (e.g. Certain features could be identified whilst watching the audio‐visual recordings). This needs to be considered when interpreting results, however, is unlikely to have caused any researcher bias as only standardized pre‐defined outcomes were assessed (e.g., presence of nursing staff, number of interruptions, duration, accuracy of notes). In future work using the Sterile Cockpit assessing for patient outcomes, an important consideration would be to ensure that a separate blinded reviewer extracts patient outcomes.

Another potential limitation of the study design was the grouping of the formal consultant‐led ward round (Wednesdays), with the everyday working ward round (all other days). This was done to ensure data were representative of the real‐world ward rounds occurring in the hospital being studied. It is possible that the effectiveness of the intervention may vary when ward rounds are consultant led compared to non‐consultant led. This variable was not controlled for in this analysis, however, a comparable number of consultant rounds were included in both the “Standard Care” cohort (22/71) and the “Sterile Cockpit” cohort (20/70), maintaining validity of comparative analysis between study arms. Further research would be needed to determine if seniority of the individual leading the ward round impacted the efficacy of the Sterile Cockpit. This could be extended further by also assessing the impact of the individual leading (regardless of their seniority).

One important, yet unavoidable limitation that should also be considered when interpreting results is the potential for bias between groups due to the significantly higher proportion of female staff leading the ward round in the “Sterile Cockpit” group. Although it could not be controlled, the potential impact of different team leaders on results should be considered when interpreting intervention outcomes. Additionally, this study was conducted in a single‐center site using an observational cohort, which needs to be considered in terms of generalizability of the results. Due to the nature of this study, a randomized control trial could not be conducted, and certain patients had to be excluded (e.g., patients held in isolation rooms). However, it is likely that this limitation did not impact results, as only ward round processes were measured, not specific patient outcomes. To reduce sampling bias in the future, stratified sampling could be utilized. To strengthen the generalizability of results, this study should be conducted multi‐center across both rural and urban areas, for both surgical and non‐surgical healthcare settings. With a larger cohort across multiple sites, this would allow the possibility to control for the sex of the leading surgeon.

The positive and measurable results in this study are promising, however it is important to consider the potential real‐world limitations of its implementation. Although the addition of time per ward round was not found to be statistically significant, it may have been clinically significant. In an environment that is already time pressured, it may not be feasible or realistic to start the rounds earlier, and this could increase hesitancy to adopt the intervention between staff. For the Sterile Cockpit to be successful in the world round, it relies on the consistent investment from a range of multi‐disciplinary members. Buy‐in from clinical leadership and heads of departments is crucial, as these members are the “team leader's” who ensure that the Sterile Cockpit is running adequately. Small additional time pressures may be a limiting factor that drives hesitancy to adopt the intervention from staff. However, the benefits of improved accuracy and timeliness of documentation, coupled with the reduced distractions and interruptions inevitably saves time throughout the day, reduces morbidity and mortality.

Although this was not specifically measured in this study, by maintaining more accurate and contemporaneous notes, and creating an environment of more engaged team members, there will inevitably be a reduced time burden in clarifying calls, rectification of mistakes and omissions, and will streamline the plan multi‐disciplinarily. When considering the long‐term efficacy of the Sterile Cockpit, the absence of a follow‐up period to determine adherence in everyday practice following the cessation of this study does limit the studies long‐term efficacy. Future research directions should endeavor to assess the longitudinal studies of the sustainability of the “Sterile Cockpit” to understand feasibility for implementation into everyday practice. Additionally, the “Sterile Cockpit” should be trialled in Acute Surgical Unit and other acute settings to determine feasibility.

By using adaptations of aviation principles, this innovative concept has demonstrated measurable improvements in accuracy of documentation, patient satisfaction, nurse involvement and reduction of interruptions. The results are further strengthened by the use of audio‐visual recordings for data collection, ensuring objective measures of outcomes.

Additionally, positive outcomes were demonstrated without extending ward round duration, highlighting feasibility and effectiveness. The daily ward round is practiced in every inpatient clinical specialty. While there are differences between hospitals, units, fields, or personnel, the fundamental concepts remain the same. The goal of every clinical team is to provide the best care for patients which is monitored in the daily round. The ‘Sterile Cockpit’ intervention can be conducted at a low‐cost as it does not require additional staffing, or significant infrastructure other than a sign and initial education session. This makes it attractive and scalable for widespread adoption across healthcare settings. Policy makers can implement this in healthcare delivery to optimize patient and staff outcomes, similar to the aviation style universal policy [44].

The “Sterile Cockpit” provides a moment to slow down and reflect, confirm the patient's plan, and empowers junior staff to speak up. The “Sterile Cockpit” significantly improved case note documentation—particularly key information, decisions and discharge planning—reduced interruptions and parallel conversations, improved patient satisfaction, and promoted nurse presence, all while adding no additional time for staff. Reducing interruptions and distractions during ward rounds increases the quality time patients spend with their treating team, which in turn can enhance patient engagement and strengthen therapeutic relationships. This simple, low‐cost intervention has the capacity to improve the quality of healthcare patients receive and ultimately improve patient outcomes.

## Author Contributions

**Ellie Treloar:** conceptualization, methodology, data curation, investigation, formal analysis, writing – original draft, writing – review and editing, visualization, funding acquisition, project administration, supervision. **Matheesha Herath:** conceptualization, writing – review and editing, writing – original draft, visualization, investigation, data curation, methodology, funding acquisition. **Meryl Altree:** data curation, investigation, validation, writing – review and editing, methodology. **Sam Potter:** investigation, writing – review and editing. **Matthew Penhall:** investigation, writing – review and editing. **David Walsh:** investigation, writing – review and editing. **Lauren Kennedy:** investigation, writing – review and editing. **Martin Bruening:** investigation, writing – review and editing. **Suzanne Edwards:** software, formal analysis, writing – review and editing. **Jesse D. Ey:** investigation, writing – original draft, writing – review and editing. **Emma L. Bradshaw:** conceptualization, project administration, visualization, writing – review and editing, supervision. **Guy J. Maddern:** conceptualization, data curation, funding acquisition, visualization, project administration, resources, writing – review and editing, writing – original draft, methodology.

## Ethics Statement

This study has been approved by the Central Adelaide Local Health Network Human Research Ethics Committee.

## Consent

Informed consent was obtained from all individual participants included in the study.

## Conflicts of Interest

The authors declare no conflicts of interest.

## Supporting information


Supporting Information S1

## Data Availability

The data that support the findings of this study are available from the corresponding author upon reasonable request.

## References

[1] M. Zegers , M. C. de Bruijne , B. de Keizer , et al., “The Incidence, Root‐Causes, and Outcomes of Adverse Events in Surgical Units: Implication for Potential Prevention Strategies,” Patient Safety in Surgery 5, no. 1 (2011): 13, 10.1186/1754-9493-5-13. 21599915 PMC3127749

[2] C. Vincent , G. Neale , and M. Woloshynowych , “Adverse Events in British Hospitals: Preliminary Retrospective Record Review,” BMJ 322, no. 7285 (2001): 517–519, 10.1136/bmj.322.7285.517. 11230064 PMC26554

[3] J. D. Ey , V. Kollias , M. B. Herath , et al., “Development and Validation of a Novel Tool for Identification and Categorization of Non‐Technical Errors Associated With Surgical Mortality,” British Journal of Surgery 111, no. 10 (2024): znae253, 10.1093/bjs/znae253. 39423032 PMC11488386

[4] A. A. Gawande , M. J. Zinner , D. M. Studdert , and T. A. Brennan , “Analysis of Errors Reported by Surgeons at Three Teaching Hospitals,” Surgery 133, no. 6 (2003): 614–621, 10.1067/msy.2003.169. 12796727

[5] S. O. Rogers Jr , A. A. Gawande , M. Kwaan , et al., “Analysis of Surgical Errors in Closed Malpractice Claims at 4 Liability Insurers,” Surgery 140, no. 1 (2006): 25–33, 10.1016/j.surg.2006.01.008. 16857439

[6] A. A. Ghaferi , J. D. Birkmeyer , and J. B. Dimick , “Variation in Hospital Mortality Associated With Inpatient Surgery,” New England Journal of Medicine 361, no. 14 (2009): 1368–1375, 10.1056/nejmsa0903048. 19797283

[7] P. H. Pucher , R. Aggarwal , and A. Darzi , “Surgical Ward Round Quality and Impact on Variable Patient Outcomes,” Annals of Surgery 259, no. 2 (2014): 222–226, 10.1097/sla.0000000000000376. 24263319

[8] T. McHugh , “The Reform of the Paris Hôtel Dieu,” in Hospital Politics in Seventeenth‐Century France: The Crown, Urban Elites and the Poor A. Cunningham and O. Grell , (Ashgate Publishing Company, 2007), 64–65.

[9] G. B. Risse , Mending Bodies, Saving Souls: A History of Hospitals (Oxford University Press, 1999), 1–752.

[10] J. A. O'Hare , “Anatomy of the Ward Round,” European Journal of Internal Medicine 19, no. 5 (2008): 309–313, 10.1016/j.ejim.2007.09.016. 18549930

[11] Royal College of Physicians RCoN . Modern Ward Rounds: Good Practice for Multidisciplinary Inpatient Review (Royal College of Physicians, 2021), 5–44.

[12] K. Shetty , S. X. W. Poo , K. Sriskandarajah , et al., “The Longest Way Round is the Shortest Way Home: An Overhaul of Surgical Ward Rounds,” World Journal of Surgery 42, no. 4 (2017): 937–949, 10.1007/s00268-017-4267-1. PMC5843677 29067515

[13] G. L. Creamer , A. Dahl , D. Perumal , G. Tan , and J. B. Koea , “Anatomy of the Ward Round: The Time Spent in Different Activities,” ANZ Journal of Surgery 80, no. 12 (2010): 930–932, 10.1111/j.1445-2197.2010.05522.x. 21114735

[14] O. Anderson , R. Davis , G. B. Hanna , and C. A. Vincent , “Surgical Adverse Events: A Systematic Review,” Americas Journal of Surgery 206, no. 2 (2013): 253–262, 10.1016/j.amjsurg.2012.11.009. 23642651

[15] N. Serou , L. M. Sahota , A. K. Husband , S. P. Forrest , R. D. Slight , and S. P. Slight , “Learning From Safety Incidents in High‐Reliability Organizations: A Systematic Review of Learning Tools That Could Be Adapted and Used in Healthcare,” International Journal for Quality in Health Care 33, no. 1 (2021): mzab046, 10.1093/intqhc/mzab046. 33729493 PMC8271183

[16] S. Bagnara , O. Parlangeli , and R. Tartaglia , “Are Hospitals Becoming High Reliability Organizations?,” Applied Ergonomics 41, no. 5 (2010): 713–718, 10.1016/j.apergo.2009.12.009. 20106470

[17] A. B. Haynes , T. G. Weiser , W. R. Berry , et al., “A Surgical Safety Checklist to Reduce Morbidity and Mortality in a Global Population,” New England Journal of Medicine 360, no. 5 (2009): 491–499, 10.1056/nejmsa0810119. 19144931

[18] E. C. Treloar , J. D. Ey , M. Herath , et al., “Optimizing Ward Rounds: Systematic Review and Meta‐Analysis of Interventions to Enhance Patient Safety,” British Journal of Surgery 112, no. 4 (2025): znaf041, 10.1093/bjs/znaf041. 40202092 PMC11979594

[19] E. C. Treloar , Y. Y. Ting , J. G. Kovoor , J. D. Ey , J. L. Reid , and G. J. Maddern , “Can Checklists Solve Our Ward Round Woes? A Systematic Review,” World Journal of Surgical Oncology 46, no. 10 (2022): 2355–2364, 10.1007/s00268-022-06635-5. PMC9436887 35781840

[20] E. C. Treloar , Y. Y. Ting , M. H. Bruening , et al., “Lost in Transcription—How Accurately Are We Documenting the Surgical Ward Round?,” ANZ Journal of Surgery 95, no. 5 (2025): 1005–1010, 10.1111/ans.70109. 40202286 PMC12105566

[21] L. Yip and B. Farmer , “High Reliability Organizations—Medication Safety,” Journal of Medical Toxicology 11, no. 2 (2015): 257–261, 10.1007/s13181-015-0471-2. 25812532 PMC4469723

[22] A. Gawande , “A Challenge for Practitioners Worldwide: WHO Safe Surgery Saves Lives,” Journal of Perioperative Practice 19, no. 10 (2009): 312. 19908666

[23] A. B. Haynes , T. G. Weiser , W. R. Berry , et al., “Changes in Safety Attitude and Relationship to Decreased Postoperative Morbidity and Mortality Following Implementation of a Checklist‐Based Surgical Safety Intervention,” BMJ Quality and Safety 20, no. 1 (2011): 102–107, 10.1136/bmjqs.2009.040022. 21228082

[24] World Health Organization, GWH . WHO Guidelines Approved by the Guidelines Review Committee, Global Recommendations on Physical Activity for Health, 2009), 7–124.

[25] A. M. Fore , G. L. Sculli , D. Albee , and J. U. L. I. A. Neily , “Improving Patient Safety Using the Sterile Cockpit Principle During Medication Administration: A Collaborative, Unit‐Based Project,” Journal of Nursing Management 21, no. 1 (2013): 106–111, 10.1111/j.1365-2834.2012.01410.x. 23339500

[26] H. M. Filer , B. L. Beringuel , K. M. Frato , M. K. Anthony , and P. Saenyakul , “Interruptions in Preanesthesia Nursing Workflow: A Pilot Study of Pediatric Patient Safety,” Journal of PeriAnesthesia Nursing 32, no. 2 (2017): 112–120, 10.1016/j.jopan.2015.01.016. 28343636

[27] C. B. Giddens and J. A. Blankenship , “The Red Square: A Healthcare Sterile Cockpit to Reduce Medication Errors,” NASN School Nurse 39, no. 2 (2024): 66–70, 10.1177/1942602x231196140. 37700542

[28] Royal College of Physicians RCoN . Ward Rounds in Medicine: Principles for Best Practice (Royal College of Physicians, 2012), 7–18.

[29] I. Salik and J. V. Ashurst , “Closed Loop Communication Training in Medical Simulation,” in Statpearl (StatPearls Publishing, 2025), 1–X. 31751089

[30] N. Krishnamohan , I. Maitra , and V. D. Shetty , “The Surgical Ward Round Checklist: Improving Patient Safety and Clinical Documentation,” Journal of Multidisciplinary Healthcare 12 (2019): 789–794, 10.2147/jmdh.s178896. 31571896 PMC6754526

[31] M. Zegers , M. C. de Bruijne , P. Spreeuwenberg , C. Wagner , P. P. Groenewegen , and G. van der Wal , “Quality of Patient Record Keeping: An Indicator of the Quality of Care?,” BMJ Quality and Safety 20, no. 4 (2011): 314–318, 10.1136/bmjqs.2009.038976. 21303769

[32] B. Prakash , “Patient Satisfaction,” Journal of Cutaneous and Aesthetic Surgery 3, no. 3 (2010): 151–155, 10.4103/0974-2077.74491. 21430827 PMC3047732

[33] F. Manzoor , L. Wei , A. Hussain , M. Asif , and S. I. A. Shah , “Patient Satisfaction With Health Care Services; an Application of Physician's Behavior as a Moderator,” International Journal of Environmental Research and Public Health 16, no. 18 (2019): 3318, 10.3390/ijerph16183318. 31505840 PMC6765938

[34] Australian Commission on Safety and Quality in Health Care . Patient‐Centred Care: Improving Quality and Safety Through Partnerships with Patients and Consumers (ACSQHC, 2011).

[35] T. Isaac , A. M. Zaslavsky , P. D. Cleary , and B. E. Landon , “The Relationship Between Patients' Perception of Care and Measures of Hospital Quality and Safety,” Health Services Research 45, no. 4 (2010): 1024–1040, 10.1111/j.1475-6773.2010.01122.x. 20528990 PMC2910567

[36] A. Cohn , “The Ward Round: What It Is and What It Can Be,” supplement, British Journal of Hospital Medicine 75, no. S6 (2014): C82–C85, 10.12968/hmed.2014.75.sup6.c82. 25075416

[37] A. Cohn , “Restore the Prominence of the Medical Ward Round,” BMJ 347, no. oct31 2 (2013): f6451, 10.1136/bmj.f6451. 24179033

[38] J. McElhatton and C. Drew , “Hurry Up Syndrome: Research Indicates That Many Aviation Safety Incidents Could Have Been Prevented if the Human Factors Elements of Time Pressure Had Been Better Understood,” Air Line Pilot 63, no. 9 (1994): 16–21.

[39] R. Bohmer , A. Edmondson , and G. Pisano , “Speeding up Team Learning,” Harvard Business Review 79, no. 9 (October 2001): 125–132.

[40] A. Babiker , M. El Husseini , A. Al Nemri , et al., “Health Care Professional Development: Working as a Team to Improve Patient Care,” Sudanese Journal of Paediatrics 14, no. 2 (2014): 9–16. 27493399 PMC4949805

[41] A. C. Edmondson , The Fearless Organization: Creating Psychological Safety in the Workplace for Learning, Innovation, and Growth (John Wiley & Sons, 2018), 1–256.

[42] T. Fukami , “Shared Decision Making With Psychological Safety,” Lancet 401, no. 10383 (2023): 1153–1154, 10.1016/s0140-6736(23)00344-6. 37030882

[43] B. N. Green and C. D. Johnson , “Interprofessional Collaboration in Research, Education, and Clinical Practice: Working Together for a Better Future,” Journal of Chiropractic Education 29, no. 1 (2015): 1–10, 10.7899/jce-14-36. 25594446 PMC4360764

[44] A. Veneman , G. Norton , and M. Blakey , Safety Recommendations (National Transportation Safety Board, 2004), 1–17.

