# Supplementary material for: A Simple Solution for a Complex Problem: The “Sterile Cockpit” to Improve Ward Rounds
Source: World J Surg. 2025 Sep 10;49(10):2769–76. doi: 10.1002/wjs.70074 (PMC12515027; doi:10.1002/wjs.70074)
Supplement: Supplementary file 1 — Supporting Information S1 [file WJS-49-2769-s001.docx]

The questions were:

- How would you rate your satisfaction of the interaction between yourself and the surgical team today?
- How certain are you that you understood the plan made today?
- How confident were you to ask questions about your care today?
- How confident are you that your concerns were addressed today?
- How approachable were your treating team today?
- How did you feel about the amount of time the surgical team spent at your bedside today?
- How focused were your treating team during the ward round today?
- Did you feel included when decisions were made about your treatment and care?
- Do you have any other comments on how this ward round experience could be improved for you?
